# Interactive Effects of Moss-Dominated Crusts and *Artemisia ordosica* on Wind Erosion and Soil Moisture in Mu Us Sandland, China

**DOI:** 10.1155/2014/649816

**Published:** 2014-06-01

**Authors:** Yongsheng Yang, Chongfeng Bu, Xingmin Mu, Hongbo Shao, Kankan Zhang

**Affiliations:** ^1^State Key Laboratory of Soil Erosion and Dryland Farming of the Loess Plateau, Institute of Soil and Water Conservation, Chinese Academy of Sciences and Ministry of Water Resources, Yangling, Shaanxi 712100, China; ^2^Institute of Soil and Water Conservation, Northwest A&F University, Yangling, Shaanxi 712100, China; ^3^Key Laboratory of Coastal Biology & Bioresources Utilization, Yantai Institute of Coastal Zone Research (YIC), Chinese Academy of Sciences (CAS), Yantai 264003, China

## Abstract

To better understand the effects of biological soil crusts (BSCs) on soil moisture and wind erosion and study the necessity and feasibility of disturbance of BSCs in the Mu Us sandland, the effects of four treatments, including moss-dominated crusts alone, *Artemisia ordosica* alone, bare sand, and *Artemisia ordosica* combined with moss-dominated crusts, on rainwater infiltration, soil moisture, and annual wind erosion were observed. The major results are as follows. (1) The development of moss-dominated crusts exacerbated soil moisture consumption and had negative effects on soil moisture in the Mu Us sandland. (2) Moss-dominated crusts significantly increased soil resistance to wind erosion, and when combined with *Artemisia ordosica*, this effect became more significant. The contribution of moss-dominated crusts under *Artemisia ordosica* was significantly lower than that of moss-dominated crusts alone in sites where vegetative coverage > 50%. (3) Finally, an appropriate disturbance of moss-dominated crusts in the rainy season in sites with high vegetative coverage improved soil water environment and vegetation succession, but disturbance in sites with little or no vegetative cover should be prohibited to avoid the exacerbation of wind erosion.

## 1. Introduction

Biological soil crusts (BSCs) are complex assemblages of phylogenetically and functionally diverse organisms such as macroscopic bryophytes (e.g., mosses and liverworts), lichens, microscopic archaea, bacteria, cyanobacteria, microfungi, and green algae [1, 2]. The biotic components of BSCs have a high tolerance for low moisture, extreme temperatures, and light and are therefore widely distributed in regions characterized by periodic dryness, intense heat, and strong light, which constitute over 35% of the world's terrestrial land [3, 4]. BSCs perform several functions in arid and semiarid ecosystems. For example, many studies have demonstrated that BSCs have a significant influence on rainwater infiltration [5], runoff [4], and evaporation [6] in arid and semiarid regions. Furthermore, BSCs prevent soil erosion by water [7] or wind [8], increase the possibility of vascular plant colonization [9], stabilize soil surface [10], and fix carbon on sparsely vegetated areas in arid regions [11]. In other words, BSCs are critical structural and functional components of arid and semiarid ecosystems.

In China, the desertification occurring in arid and semiarid regions threatens approximately 3.3 × 10^6^ km^2^ of land, which is inhabited by 400 million people [12]. As the main indicator of dune stabilization, BSCs have been widely distributed in arid and semiarid regions. In Mu Us sandland, BSCs have widely developed in enclosed regions and have already deeply affected soil moisture and vegetation succession [13]. However, most literatures on BSCs, hydrology, or wind erosion in China have focused mainly on arid-desert regions, such as the Tengger Desert, Kubuqi Desert, and Gurbantünggüt Desert. Little attention has been paid to the roles of BSCs in soil moisture or wind erosion in the Mu Us sandland, where the annual average rainfall is approximately 400 mm [14]. Therefore, studying the effects of BSCs on soil moisture and wind erosion in Mu Us sandland could contribute to a more complete understanding of the ecological function of BSCs.

Several previous studies have investigated the function of BSCs on water dynamics, but the results have not been consistent due to the complex process of interception by BSCs. For example, BSCs have been found to enhance [5, 15] or reduce [16] water infiltration and to increase [17] or decrease [14] the occurrence of runoff, while other studies have shown that BSCs have no effect on infiltration [18]. In opposition to these studies, other studies report that BSCs increased the soil threshold friction velocities, which are required to detach particles from the soil surfaces [11] and drastically decrease wind erosion [19]. However, BSCs are easily damaged by disturbance, such as livestock, human traffic, sand burial, and animal burrowing due to their weak structural attributes [16].* Artemisia ordosica* is one of the dominant shrubs in the Mu Us sandland [20], and BSCs often grow under it. Researchers generally agree that shrubs with dense branches (e.g.,* Artemisia ordosica*) protect soil from wind erosion [21]. At present, many researchers are studying the effects of* Artemisia ordosica* alone and moss-dominated crusts alone on wind erosion and soil moisture [11, 21, 22], respectively, but less attention has been paid to the interactive effects of BSCs and* Artemisia ordosica* on these processes. Thus, we hypothesize that moss-dominated crusts in the Mu Us sandland could reduce the infiltration depths of rainwater and decrease soil moisture in the deep layer and that the ability of moss-dominated crusts under* Artemisia ordosica* to resist wind erosion is significantly lower than that of moss-dominated crusts alone. If true, moderate disturbance to moss-dominated crusts under vegetation could improve soil moisture while maintaining wind erosion control. These actions would positively benefit ecological restoration in arid and semiarid regions.

The objectives of this research are as follows: (1) to quantify the effects of moss-dominated crusts on soil moisture and wind erosion, and (2) to study the interactive effects of moss-dominated crusts and* Artemisia ordosica* in the Mu Us sandland on soil moisture and wind erosion. Based on these analyses, we discuss the necessity and feasibility of disturbance on moss-dominated crusts in semiarid regions.

## 2. Materials and Methods

### 2.1. Experiment Site

A field experiment was conducted in Gechougou located in Shenmu County Shaanxi province (38°10′–39°05′ N latitude, 109°40′–110°30′ E longitude), China. The average annual temperature in this region is 7.8°C, and the average temperatures of the hottest month (July) and the coldest month (January) are 23.9°C and −9.8°C, respectively. The prevailing wind is from the northwest, and the annual average wind speed is 3.2 m·s^−1^, with a maximum wind speed of 24 m·s^−1^ and more than 200 days per year having a wind speed greater than 5 m·s^−1^ [23]. The average annual rainfall and evaporation are 440.8 mm and 2090 mm, respectively. The dominant soil type is aeolian sandy soil and the landscape is characterized by mobile and semifixed sand dunes. The major vegetation consists of drought-tolerant, short shrubs and grasses, including* Artemisia ordosica, Salsola passerina, *and* Caragana microphylla* [20]. The annual mean hours of sunshine are 2800–3100 hours, and the annual total solar radiation is 138–150 kcal cm^−2^ [24]. The groundwater level is low and small lakes called “Haizi” are widely distributed in this region. BSCs in the experiment site are dominated by moss, while algae are less prevalent. The species of BSCs that have been identified are* Bryum pallescens*,* Bryum recurvulum *Mitt,* Bryum argenteum,* and* Barbula unguiculata *Hedw. The Mu Us sandland is the largest mobile dune system of the dry and nutrient-poor grasslands in northwestern of China, where desertification is becoming problematic [25].

### 2.2. Method

#### 2.2.1. Experimental Design

**Figure 1:**
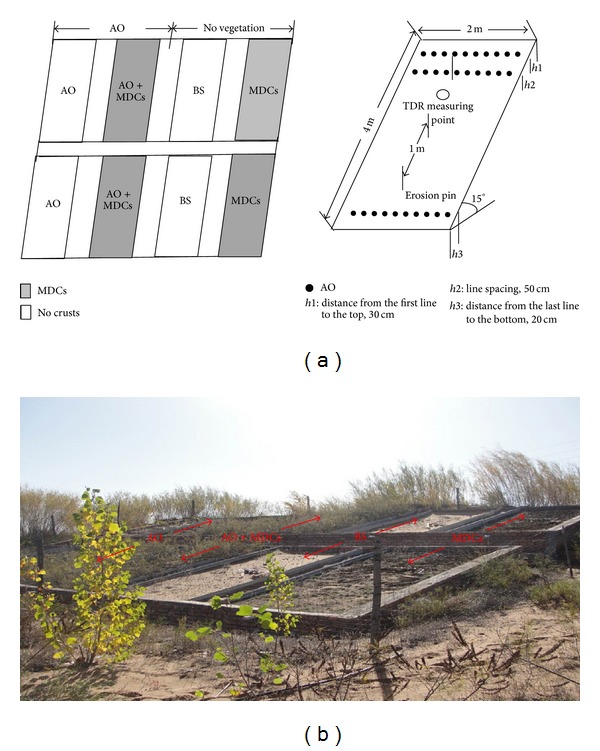
Sketch map (a) and photograph (b) of the 8 plots.

In mid-April 2008, eight experimental plots were established on a 15° slope. Each plot was 4 m × 2 m in size and oriented from south to west.* Artemisia ordosica *was planted in four plots in May 2008, and seedlings were thinned into a rectangular shape (30 cm × 50 cm) after the seeds germinated. Four separate plots were created with bare sand. In July 2008, well-developed moss-dominated crusts from another research site were carefully translocated to the soil surface of two bare sand plots and two* Artemisia ordosica* plots. The experiment included four treatments: moss-dominated crusts (MDCs) alone,* Artemisia ordosica* (AO) alone, bare sand (BS), and* Artemisia ordosica* combined with moss-dominated crusts (AO + MDCs). Each treatment had two replicates (Figure 1). Fiberglass access tubes (inner diameter: 40 mm; length: 250 cm) were installed in the middle of each plot. Weeds were cleaned regularly and well managed.

In October 2010, the vegetative cover and BSCs were 50% and 95%, respectively. The thickness of crusts measured using a Vernier caliper was 1.61 ± 0.10 cm (means ± SE, *n* = 12). The soil particle size distribution at 0–2 cm of BS and MDCs was measured by Mastersizer 2000E (Malvern Instruments Ltd. Worcestershire, UK) [26], shown in Table 1.

#### 2.2.2. Soil Moisture and Wind Erosion Measurement

**Figure 2:**
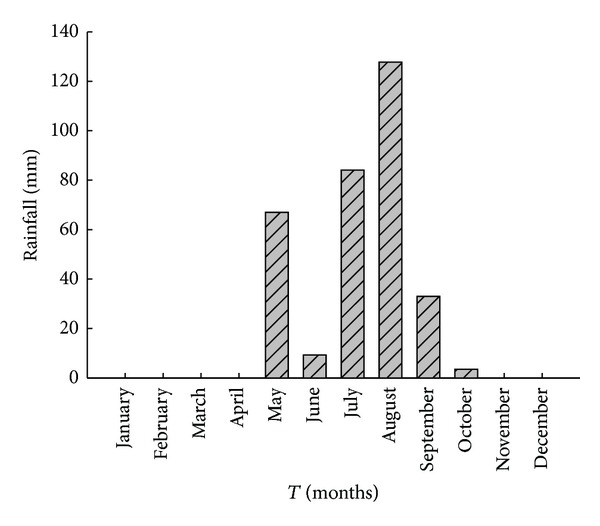
Monthly rainfall in the study region in 2011.

Soil moisture monitoring was conducted from April to September in 2011 (in the fourth year after layout). The soil moisture content at 0–16 cm was collected using a Probe-TDR (TRIME-IPH) (IMKO, Ettlingen, Germany), and the soil moisture content at 20–240 cm (at 10 cm intervals from 20 to 100 cm and 20 cm intervals from 100 to 240 cm) was collected at each plot using a Tube-TDR [27]. The soil moisture content was measured three times from April to May and eight times from July to September. Soil moisture was collected 24 hours before and after rainfall events, according to the weather forecasts. Rainfall data during the experiment were collected using a tipping-bucket rain gauge (Figure 2).

In October 2010, the erosion pins, which were 50 cm (30 cm above soil surface) in height, were inserted at upper, middle, and lower sites of each plot (Figure 1), with the notch of the erosion pin flush with the soil surface. The height from the notch on the erosion pin to the soil surface in October 2011 was measured. The annual wind erosion was calculated using the following equation [28]:

(1) $$\begin{array}{c} Q=\frac{H\times 1.5\times 10^{-6}}{10^{-8}}, \end{array}$$

where *Q* is the annual wind erosion in t/ha/a, *H* is the change in height between notches on the erosion pin and soil surface in one year in cm/a, 1.5 is the bulk density of soil in g/cm^3^ [28], 10^−6^ is the conversion between gram and ton, and 10^−8^ is the conversion between square centimeter and hectare.

### 2.3. Statistical Analysis

Data were expressed as the means ± standard error. Significant differences among different treatments were tested by one-way ANOVA and LSD using SPSS 12.0 (SPSS Inc., Chicago, IL, USA). Significance was set at *P* < 0.05.

## 3. Results

### 3.1. Effects of Moss-Dominated Crusts on Infiltration

**Figure 3:**
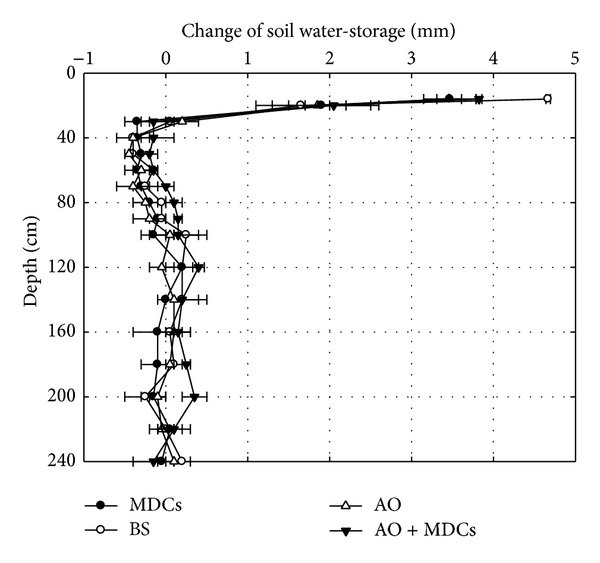
Change in soil water-storage of moss-dominated crusts plots (MDCs), bare sand plots (BS),* Artemisia ordosica* plots (AO), and* Artemisia ordosica *combined with moss-dominated crusts plots (AO + MDCs) after an 8.3 mm rain event.

**Figure 4:**
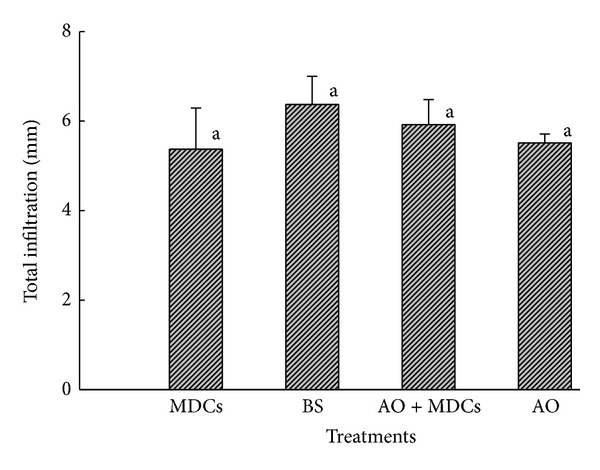
Total infiltration of the moss-dominated crusts plots (MDCs), bare sand plots (BS),* Artemisia ordosica* plots (AO), and* Artemisia ordosica* combined with moss-dominated crusts plots (AO + MDCs) after an 8.3 mm rain event. Note: different letters indicate significant differences at a 5% probability level.

The variation in soil water-storage and the total infiltration at different soil depths for the four plots after an 8.3 mm rain event are shown in Figures 3 and 4, respectively. The results indicated that the infiltration depths of MDCs alone and AO + MDCs were all lower than those of AO alone and the BS plots (Figure 3). For the total infiltration (sum of increased soil moisture), there was no significant difference among the four plots (Figure 4). These results indicate that when the daily rainfall is 8.3 mm, the existence of MDCs reduces the infiltration depth of rainwater and retains rainwater in shallower soil.

### 3.2. Effects of Moss-Dominated Crusts on Profile Distribution of Soil Moisture

**Figure 5:**
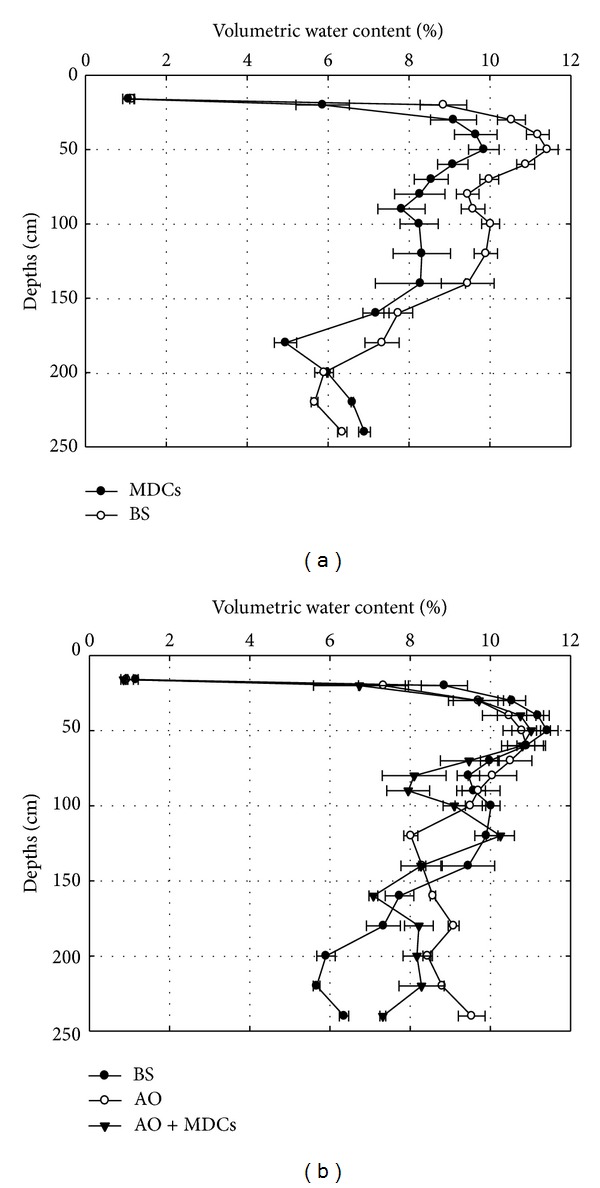
Volumetric water content from 10 cm to 240 cm of bare sand plots (BS), moss-dominated crusts plots (MDCs),* Artemisia ordosica* plots (AO), and* Artemisia ordosica* combined with moss-dominated crusts plots (AO + MDCs) in the dry season (April to May).

**Figure 6:**
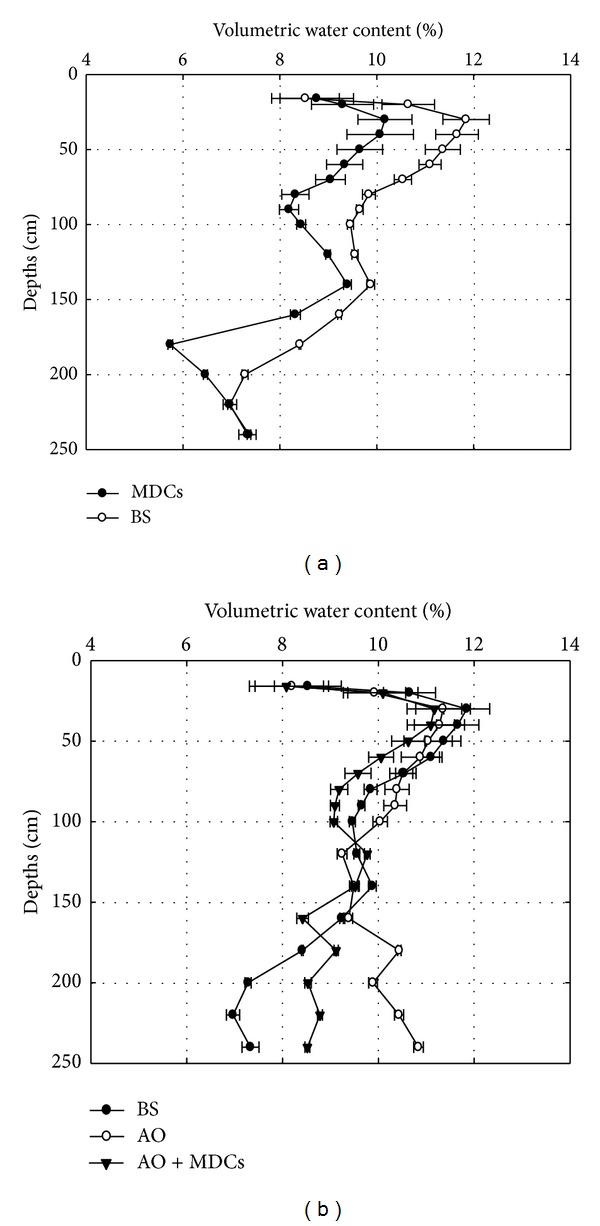
Volumetric water content from 10 cm to 240 cm of bare sand plots (BS), moss-dominated crusts plots (MDCs),* Artemisia ordosica* plots (AO), and* Artemisia ordosica *combined with moss-dominated crusts plots (AO + MDCs) in the rainy season (July to September).

The profiles of the distribution of soil moisture from 0 cm to 240 cm in the dry (April to May) and rainy (July to September) seasons in each plot are shown in Figures 5 and 6. In both the dry and rainy seasons, most of the soil moisture content observed for the four plots was below 12%.

In the dry season (Figure 5(a)), the soil moisture content in depths above 200 cm in the MDCs alone plots was lower than the content in the BS plots, while an opposite trend was observed in depths below 200 cm. In the rainy season (Figure 6(a)), the effects of MDCs on soil moisture were similar to those observed in the dry season, except the soil moisture content at a depth of 0–16 cm in the MDCs alone plots was higher than that in the BS plots. These results suggest that the presence of moss-dominated crusts alone could retain the rainwater in shallow soil during the rainy season.

Figure 5(b) shows that the soil moisture contents in the depth ranges of 0–50 cm and 100–140 cm in the AO alone plots were lower than those in the BS plots and that the soil moisture content from 0 to 70 cm in the AO alone plots was lower than that in the BS plots in the rainy season (Figure 6(b)). However, in both the dry and rainy seasons, the soil moisture content from 0 to 120 cm in AO + MDCs plots was always lower than that in the BS plots. Meanwhile, compared with the AO alone plots, the average soil moisture content from 0 to 240 cm in the AO + MDCs plots decreased by 5.7% and 7.5% in both the dry and rainy seasons. These results suggest that the development of MDCs under AO not only increased the depth of soil moisture consumption but also led to greater losses of soil moisture.

The average soil moisture content at different depths, the efficiency of reducing the soil moisture content at depth of 0–160 cm (160 cm is the length of a three-year-old AO root [29]), and the contributions of MDCs to decreasing soil moisture following the different treatments during the dry and rainy seasons are listed in Tables 2 and 3, respectively. The results showed the following: (1) regardless of whether the plot had AO coverage, MDCs had no significant effects on soil moisture content from 0 to 16 cm (*P* > 0.05); (2) during both the dry (Table 2) and rainy (Table 3) seasons, the efficiency of reducing soil moisture content from 0 to 160 cm in each plot was found to decrease in the following order: MDCs alone > AO + MDCs > AO alone, and the difference between the soil moisture content in each treatment was statistically significant (*P* < 0.05); and (3) the average soil moisture content in the deep layer (160–240 cm) of the AO alone plots increased by 11.1% and 16.3% compared with that of the AO + MDCs plots in the dry and rainy seasons, respectively. The difference between the treatments was significant (*P* < 0.05). To distinguish the contributions of MDCs from those of MDCs planted with AO on the reduction of soil moisture, the contribution of MDCs to reduction of soil moisture content was analyzed further. The analysis showed that compared with the BS plots, the AO + MDCs plots decreased the soil moisture from 0 to 160 cm depth in the dry and rainy seasons by 8.4% and 5.7%, respectively, and the proportional contribution of MDCs was 46.4% and 82.5%, respectively. 

### 3.3. Effects of Moss-Dominated Crusts on Wind Erosion

The annual wind erosion of each plot in 2011 is listed in Table 4. The presence of MDCs or AO significantly reduced wind erosion (*P* < 0.05). The contributions of the different treatments to reduce annual wind erosion were found to decrease in the following order: AO + MDCs > MDCs alone > AO alone. It is worth noting that MDCs alone reduced wind erosion by up to 90.6%, but the contribution of MDCs to the reduction of wind erosion dramatically dropped to 21.3% when combined with AO. These results indicate that an appropriate disturbance of MDCs could prevent drastic increases in wind erosion in those sites where vegetation coverage has reached a relatively high degree (>50%). This conclusion is similar to that found in the Tengger Desert [30].

## 4. Discussion and Conclusion

### 4.1. How Do Moss-Dominated Crusts Affect Soil Moisture?

Soil moisture plays an important role in soil nutrient cycling, soil temperature, and vegetation distribution [31–34]. The results of our study showed that moss-dominated crusts reduced the water infiltration depths and retained rainwater in shallow soils and these results were similar to those found in previous studies [16, 22]. This phenomenon could be explained by three factors: (1) BSCs in the Mu Us sandland were not water repellent and they can absorb a large amount of water [14]; (2) the formation of BSCs on the surface of sand dunes caused a decrease in soil particle size [35], and the water-holding capacity of subsurface soil was largely enhanced [36]; (3) during rainfall events, dust that had fallen on the crusts and swelled microbial exudates (e.g., extracellular polymeric substances) sealed the matrix porosity of BSCs [37, 38] and prolonged the time that water remained on the surface of BSCs [14]. A study in the Tengger Desert found that BSCs reduced the rainwater infiltration depths when the daily rainfall was below 10 mm [39], which is supported by the results presented in this study. Based on the long-term monitoring of rainfall in the Mu Us sandland, researchers found that 84.6% of the daily rainfall over the entire year was less than 10 mm for this region [13]. Thus, moss-dominated crusts in the Mu Us sandland could reduce the infiltration depths of most rainfall events and retain rainwater in shallow soil. However, crusts resulted in a greater total water loss through evaporation under abundant precipitation (high soil moisture) [40, 41]. Soil moisture that was retained in shallow soil could be evaporated quickly. At the end of the rainy season, the soil moisture in soil covered by moss-dominated crusts was lower than that in bare sand. Therefore, in the long run, moss-dominated crusts in the Mu Us sandland have negative effects on soil moisture and are harmful for the succession of deep-rooted plants.

### 4.2. Necessity and Feasibility of Disturbance of Moss-Dominated Crusts 

Previous research indicates that BSCs play a positive role in the initial stage of their growth, such as improving the characteristics of soil's physical structure and chemical properties [42], enriching shallow soil [43], and promoting the germination and colonization of herbaceous plants [44]. However, there was an opposite trend in the later period of BSCs development. First, thick and hard moss-dominated crusts act as a “coat” and prevent the seeds of perennial plants from penetrating into the soil [45, 46], reduce the number of juvenile plants, and cause imbalances in the age-class distribution of plant populations. Second, the growth of moss-dominated crusts increased the loss of soil moisture and significantly reduced soil moisture in deeper soil. Therefore, the further development of moss-dominated crusts is harmful for the normal succession of deep-root vegetation and has negative effects on the recovery process of degraded ecosystem in arid and semiarid regions.

Our findings suggest that when the vegetative cover has reached a relatively high degree (>50%), a disturbance of moss-dominated crusts will not drastically increase wind erosion. Research in the Tengger Desert has shown that the disturbance of moss-dominated crusts increases the amount and depth of rainfall infiltration [17] and decreases the evaporation rate [16], which is of great benefit to improving the soil moisture in arid and semiarid regions. The growth period of* Artemisia ordosica* in the Mu Us sandland begins in mid-March and is most vigorous in July. During this period, wind speeds decreased, while rainfall increased gradually. Thus, we conclude that in sites with high vegetative cover, appropriate disturbance measurements should be conducted on moss-dominated crusts in late April or early May (at the end of the wind season and at the start of the rainy season). In this case, the disturbance of moss-dominated crusts under vegetation can improve soil moisture to some degree, which is of benefit to the growth of the vegetation and will not significantly increase soil erosion. However, in sites with little or no vegetation, moss-dominated crusts primarily function in wind-breaking and sand fixation, and an improper disturbance would greatly increase the occurrence of desertification. Thus, disturbance should be strictly prohibited in these regions. It is necessary to note that we studied the effects of severe disturbance, which completely removed the moss-dominated crusts under vegetation, on soil moisture and wind erosion and that these effects are likely different from the effects of slight or intermediate disturbance. Thus, to comprehensively understand the interactive effects of different disturbance degree on BSCs on soil moisture and wind erosion, more field studies are needed.

## Figures and Tables

**Table 1:** Soil particle size of the top layer (0–2 cm) in bare sand (BS) and moss-dominated crusts (MDCs).

	<0.002 mm	0.002–0.02 mm	0.02–0.2 mm	0.2–2 mm
MDCs	0.7%	2.0%	35.1%	62.2%
BS	0.3%	1.1%	12.5%	86.1%

**Table 2:** Average soil water content of moss-dominated crusts alone plots (MDCs), bare sand plots (BS), *Artemisia ordosica* alone plots (AO), and *Artemisia ordosica* combined with moss-dominated crusts plots (AO + MDCs) at different depths in the dry season.

Treatment plot	MDCs	BS	AO + MDCs	AO
0–16 cm	1.1 ± 0.1^ab^	1.2 ± 0.1^a^	0.9 ± 0.1^b^	0.9 ± 0.0^ab^
20–40 cm	8.2 ± 0.6^a^	10.2 ± 0.4^a^	9.1 ± 0.8^a^	9.2 ± 0.6^a^
40–160 cm	8.4 ± 0.5^a^	9.8 ± 0.3^b^	9.1 ± 0.4^ab^	9.6 ± 0.3^b^
160–240 cm	6.1 ± 0.1^b^	6.3 ± 0.1^bc^	8.0 ± 0.3^d^	9.0 ± 0.1^a^
0–160 cm ASW (%)	7.8 ± 0.2^b^	9.2 ± 0.2^c^	8.5 ± 0.2^ab^	8.8 ± 0.2^ac^
0–160 cm ERS (%)	15.7 ± 1.3^a^	0.0^b^	8.4 ± 0.5^c^	4.5 ± 1.6^d^
0–160 cm CBR (%)	15.7 ± 1.3^a^		3.9 ± 1.4^b^	

Note: different letters in the same column indicate significant differences at 5% probability level.

Average soil moisture content, ASW.

Efficiency of reducing soil moisture content = (1 − soil moisture content in current treatment/soil moisture content in BS) × 100, ERS.

Contribution of MDCs to reducing soil moisture content = ((1 − soil moisture content in current treatment with MDCs/soil moisture content in BS) − (1 − soil moisture content in current treatment without MDCs/soil moisture content in BS)) × 100, CBR.

**Table 3:** The average soil water content of moss-dominated crusts alone plots (MDCs), bare sand plots (BS), *Artemisia ordosica* alone plots (AO), and *Artemisia ordosica* combined with moss-dominated crusts plots (AO + MDCs) at different depths in the rainy season.

Treatment plot	MDCs	BS	AO + MDCs	AO
0–16 cm	8.8 ± 0.8^a^	8.5 ± 0.7^a^	8.1 ± 0.8^a^	8.2 ± 0.8^a^
20–40 cm	9.8 ± 0.5^a^	11.4 ± 0.4^b^	10.8 ± 0.5^ab^	10.9 ± 0.5^ab^
40–160 cm	8.9 ± 0.2^a^	10.1 ± 0.1^b^	9.5 ± 0.1^c^	10.2 ± 0.1^bd^
160–240 cm	6.6 ± 0.0^a^	7.5 ± 0.1^b^	8.7 ± 0.0^c^	10.4 ± 0.1^d^
0–160 cm ASW (%)	9.1 ± 0.2^a^	10.3 ± 0.1^b^	9.7 ± 0.2^c^	10.2 ± 0.2^b^
0–160 cm ERS (%)	11.5 ± 1.0^a^	0.0^b^	5.7 ± 0.4^c^	1.0 ± 0.6^b^
0–160 cm CBR (%)	11.5 ± 1.0^a^		4.7 ± 0.4^b^	

Note: different letters in the same column indicate significant differences at 5% probability level.

Average soil moisture content, ASW.

Efficiency of reducing soil moisture content = (1 − soil moisture content in current treatment/soil moisture content in BS) × 100, ERS.

Contribution of MDCs to reducing soil moisture content = ((1 − soil moisture content in current treatment with MDCs/soil moisture content in BS) − (1 − soil moisture content in current treatment without MDCs/soil moisture content in BS)) × 100, CBR.

**Table 4:** Annual wind erosion of moss-dominated crusts alone plots (MDCs), bare sand plots (BS), *Artemisia ordosica* alone plots (AO), and *Artemisia ordosica* combined with moss-dominated crusts plots (AO + MDCs) in 2011.

Treatment plot	Annual wind erosion (t·ha^−1^·a^−1^)	Efficiency of reducing wind erosion (%)	Contribution of MDCs to reducing annual wind erosion (%)
MDCs	51.5 ± 7.0^bc^	90.6 ± 3.2^a^	90.6 ± 3.2^a^
BS	592.5 ± 132.5^a^	0.0^c^	
AO + MDCs	18.5 ± 10.0^b^	96.3 ± 2.5^a^	21.3 ± 7.5^b^
AO	135 ± 26.0^c^	75.0 ± 10.0^b^	

Note: different letters in the same column indicate significant differences at 5% probability level.

Efficiency of reducing wind erosion = (1 − annual wind erosion in current treatment/annual wind erosion in BS) × 100.

Contribution of MDCs to reducing wind erosion = ((1 − annual wind erosion in current treatment with MDCs/BS) − (1 − annual wind erosion in current treatment without MDCs/BS)) × 100.
